# Oral administration of monogalactosyl diacylglycerol from spinach inhibits colon tumor growth in mice

**DOI:** 10.3892/etm.2012.792

**Published:** 2012-11-01

**Authors:** NAOKI MAEDA, YASUO KOKAI, TAKAHIKO HADA, HIROMI YOSHIDA, YOSHIYUKI MIZUSHINA

**Affiliations:** 1 Laboratory of Food and Nutritional Sciences, Department of Nutritional Science, Kobe-Gakuin University, Kobe, Hyogo 651-2180;; 2 Department of Biomedical Engineering, Sapporo Medical University School of Medicine, Sapporo 060-8556;; 3 Hada Giken Co. Ltd., Yamaguchi 753-0047;; 4 Cooperative Research Center of Life Sciences, Kobe-Gakuin University, Kobe, Hyogo 651-8586, Japan

**Keywords:** monogalactosyl diacylglycerol, γ-cyclodextrin, DNA polymerase, antitumor, anti-proliferation

## Abstract

Previously, we observed that purified monogalactosyl diacylglycerol (MGDG), a major glycoglycerolipid from spinach, selectively inhibits the activities of mammalian replicative DNA polymerases (α, δ and ε). However, the function of MGDG following ingestion is not well-known. In the present study, spinach MGDG suppressed the proliferation of Colon26 mouse colon cancer cells with an LD_50_ of 24 *μ*g/ml *in vitro*. γ-cyclodextrin (CD)-MGDG complex was prepared and administered orally following Colon26 mouse tumor adhesion for 26 days. It was observed that 20 mg/kg equivalent (eq.) of the CD-MGDG complex reduced tumor volume by ∼60% compared with that of the vehicle-treated controls. In immunohistochemical analysis, the CD-MGDG complex group showed a decreased number of proliferating cell nuclear antigen (PCNA)-positive cells and reduction of mitosis in the tumor cells compared with the control group. In addition, the CD-MGDG complex increased the number of terminal deoxynucleotidyl transferase dUTP nick-end labeling (TUNEL)-positive apoptotic cells and inhibited CD31-positive tumor blood vessel growth significantly. These results suggest that MGDG has the potential for cancer prevention and health promotion.

## Introduction

Cancer is a major public health problem worldwide. Epidemiological and animal studies indicate that the consumption of vegetables and fruits with natural chemopreventive agents, alone or in a mixture, is associated with a reduced risk of cancer development (1–3).

The human genome encodes at least 15 DNA polymerases that perform cellular DNA synthesis (4,5). Eukaryotic cells contain three replicative DNA polymerases (α, δ and ε), mitochondrial DNA polymerase γ and at least eleven repair-related DNA polymerases [β, ζ, η, θ, ι, κ, λ, *μ*, ν, terminal deoxynucleotidyl transferase (TdT) and REV1] (4–6). Targeted DNA polymerase inhibition exerts an antitumor effect, since replicative DNA polymerases are essential for cancer cell growth (5). As a result of screening for DNA polymerase inhibitors, we observed that monogalactosyl diacylglycerol (MGDG) and sulfoquinovosyl diacylglycerol (SQDG) inhibit replicative DNA polymerase activity (7–10). Higher plants contain, particularly within the thylakoid membranes of the chloroplast, major glycoglycerolipids, including MGDG, SQDG and digalactosyl diacylglycerol (DGDG) (11). It is known that glycoglycerolipids are contained in vegetables, fruits and grains (12,13), and we observed that spinach was the most abundant source of glycoglycerolipids of the vegetables tested (14). Therefore, to examine the glycoglycerolipid properties, we purified these three glycoglycerolipids from spinach and observed that MGDG was obtained in the highest amount (15). MGDG purified from spinach also inhibited replicative DNA polymerases. However, it had no inhibitory effect on the other DNA polymerases tested (repair type-DNA polymerases β, η, ι, κ, λ and *μ*). DGDG did not influence all mammalian DNA polymerases, whereas SQDG inhibited the activities of all (both replicative and repair-related) mammalian DNA polymerases (7).

MGDG has been shown to exert bioactive effects on cells and animals, including cancer cell growth inhibition (7) and anti-angiogenesis (16,17) and anti-inflammatory activity (18). However, the inhibition of tumor growth *in vivo* following the oral administration of MGDG has not been evaluated. MGDG has characteristic high viscosity and low solubility; γ-cyclodextrin (CD) was used to solve this problem. In the present study, we investigated the bioactivity, particularly the antitumor activity, of orally administered spinach MGDG mixed with CD (CD-MGDG complex). In addition, we discuss the effects of the CD-MGDG complex on proliferation and angiogenesis in colon tumors that were observed using a histopathological technique. We evaluated the antitumor effects of orally administered MGDG in mice to develop a food-derived anticancer compound.

## Materials and methods

### Preparation of CD-MGDG complex

**Figure 1:**
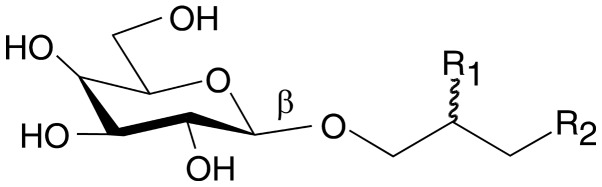
Chemical structure of monogalactosyl diacylglycerol (MGDG). R_1_ and R_2_ are acyloxy groups (derived from fatty acids).

MGDG was purified from dried spinach (*Spinacia oleracea* L.) as described previously (7). An MGDG purification grade of >98% was used in this study. The chemical structure of MGDG is shown in Fig. 1 and the composition of the acyloxy groups of MGDG (R_1_ and R_2_ in Fig. 1) has been described previously (7). MGDG (50 mg) was dissolved in 2.5 ml ethanol and a CD (Wako Pure Chemical Industries, Osaka, Japan) solution (500 mg/2.5 ml in distilled water) was added. The mixture of MGDG and CD was homogenized by mixing at 2500 rpm for 30 min at room temperature. After incubation overnight at room temperature in the dark, the mixture of MGDG and CD was freeze-dried in a vacuum at −50°C overnight.

### In vitro anti-cell proliferation activity

The mouse colon adenocarcinoma cell line, Colon26, was provided by the Cell Resource Center for Biomedical Research (Tohoku University, Sendai, Japan). The Colon26 cells were cultured in RPMI-1640 (Wako Pure Chemical Industries) supplemented with 10% fetal bovine serum (Equitech-Bio, Kerrville, TX, USA), penicillin (100 U/ml, Nacalai Tesque, Kyoto, Japan) and streptomycin (100 *μ*g/ml, Nacalai Tesque). The cells were cultured in an atmosphere of 95% air and 5% CO_2_ at 37°C. Cell proliferation was measured by the 3-(4,5-dimethylthiazol-2-yl)-2,5-diphenyl-2H-tetrazolium bromide (MTT; Sigma-Aldrich, St. Louis, MO, USA) assay (19). The Colon26 cells were trypsinized and plated in a 96-well plate at 5,000 cells per well (n=5) in medium overnight. Following cell adhesion, MGDG (0–50 *μ*M) was added to the Colon26 cells. After 24 h, MTT solution was added to all wells and the cells were incubated for 4 h at 37°C. The medium containing MTT was removed and dimethyl sulfoxide (DMSO) was added to each well to dissolve formazan crystals. The absorbance of each well was measured using a microplate reader (Vmax; Molecular Devices, Osaka, Japan) at test and reference wavelengths of 570 and 630 nm, respectively.

### In vivo assessment of antitumor activity

Five-week-old specific pathogen-free female Balb/c mice were provided by Japan SLC (Shizuoka, Japan). The mice were fed a standard diet (MF; Oriental Yeast Co., Ltd., Osaka, Japan) and had free access to water. The present study was approved by the Kobe-Gakuin University Animal Committee according to the guidelines for the ‘Care and Use of Laboratory Animals’ of the University.

Following one week of breeding, s.c. (hypodermic injection) tumors were induced by the inoculation of 1×10^6^ Colon26 cells s.c. into the Balb/c mice. The tumor-bearing mice were divided randomly into three groups and treatment was initiated with the CD-MGDG complex or vehicle control (CD alone) 5 days after tumor inoculation, when the tumors had achieved a tumor volume [tumor volume = length × (width)^2^ × 0.5] of 25–50 mm^3^. The CD-MGDG complex groups (44 or 220 mg/kg; n=5 or 6, respectively) were treated orally (p.o.) with 4 or 20 mg/kg equivalent (eq.) of MGDG, daily for 26 days. The control mice received CD alone p.o. (200 mg/kg; n=6) daily prior to examination.

When the treatment was completed, all mice were examined at necropsy for gross organ abnormalities. The lungs, heart, spleen, stomach, liver, pancreas, kidney, intestine and brain were collected, fixed in 10% formalin, and embedded in paraffin for histopathological evaluation with hematoxylin and eosin (H-E) staining. The tumors were also embedded in paraffin for H-E and proliferating cell nuclear antigen (PCNA) staining. The remainder of the tumor was embedded in optimal cutting temperature (OCT) compound (Sakura Finetek Japan, Tokyo, Japan) for histopathological evaluation by CD31 staining and a terminal deoxynucleotidyl transferase dUTP nick-end labeling (TUNEL) assay.

### Assessment of tumor cell proliferation

Tumor cell proliferation was measured using the mitotic index and PCNA expression with H-E and immunohistochemical staining. Sections (3 *μ*m) were deparaffinized in xylene and alcohol, and transferred to phosphate-buffered saline (PBS). The deparaffinized sections were stained with H-E to calculate the number of mitotic cells. The mitotic index [number of cells in mitosis/high power field (HPF)] of five random non-necrotic fields at ×400 magnification was determined. The remaining deparaffinized sections were stained with PCNA monoclonal antibody (sc-53; Santa Cruz Biotechnology, Inc., Santa Cruz, CA, USA) to evaluate cell proliferation. Sections (3 *μ*m) were incubated with PCNA antibody (1:500 dilution) overnight at 4°C. The sections were then rinsed three times with PBS for 5 min each and the slides were incubated with a secondary goat anti-mouse antibody conjugated to peroxidase (Nichirei Biosciences, Tokyo, Japan) for 10 min at room temperature. The sections were washed three times with PBS for 5 min. Positive reactions were rendered visible by incubating the slides with 3,3′-diaminobenzidine (DAB; Nichirei Biosciences) for 5 min at room temperature. The sections were rinsed with distilled water, counterstained with hematoxylin for 5 sec and mounted. To quantify PCNA expression, the number of positive cells was counted in 3–5 fields at ×400 magnification.

### Immunofluorescence staining for CD31

Fresh frozen tissues were cut into 10-*μ*m sections and mounted on positively charged slides. The sections were fixed in cold methanol/acetone (1:1) for 10 min and then washed three times with PBS for 5 min each time. The slides were placed in a humidified chamber and incubated with 5% goat serum for 20 min at room temperature. The sections were then incubated overnight with a 1:400 dilution of rat anti-mouse CD31 antibody (BD Pharmingen, San Diego, CA, USA) at 4°C. The sections were then rinsed three times with PBS for 5 min each and the slides were incubated with a secondary goat anti-rat IgG antibody conjugated to Alexa 594 (1:500 dilution; Invitrogen Japan K.K., Tokyo, Japan) for 10 min at room temperature. The slides were then washed three times with PBS for 5 min each, mounting medium was placed on each slide and the slides were covered with glass coverslips. To quantify CD31 expression, five fields at ×250 magnification were examined for each tumor with a confocal laser scanning microscope (LSM 510 META, Carl Zeiss MicroImaging, Tokyo, Japan). The fluorescence images were analyzed using ZEN software (Carl Zeiss MicroImaging).

### TUNEL assay

The TUNEL assay was performed using an Apoptosis Detection kit (Takara Bio, Shiga, Japan). Fresh frozen sections were fixed with 4% paraformaldehyde for 30 min at 4°C and then washed with PBS for 30 min. The sections were incubated with 0.3% H_2_O_2_ in methanol for 30 min to block endogenous peroxidase and then washed three times with PBS for 5 min each. The sections were permeabilized with permeabilization buffer on ice for 5 min. The slides were placed in a humidified chamber and incubated with TdT enzyme including fluorescein isothiocyanate (FITC)-conjugated dUTP for 60 min at 37°C. The slides were washed three times with PBS for 5 min. The sections were incubated with anti-FITC HRP conjugate for 30 min at 37°C and the slides were washed three times with PBS for 5 min. Positive reactions were rendered visible by incubating the slides with DAB for 10 min at room temperature. The sections were rinsed with distilled water, conterstained with hematoxylin for 5 sec and mounted. To quantify TUNEL-positive expression, the number of positive cells was counted in five fields at ×400 magnification.

### Statistical analysis

All experiments show the mean ± SE between groups. Comparisons were made using the Mann-Whitney U test or Steel’s test using the KyPlot 5.0 software package (LyensLab, Tokyo, Japan). P<0.05 was considered to indicate a statistically significant result.

## Results

### Effect of spinach MGDG on in vitro cancer cell proliferation

**Figure 2:**
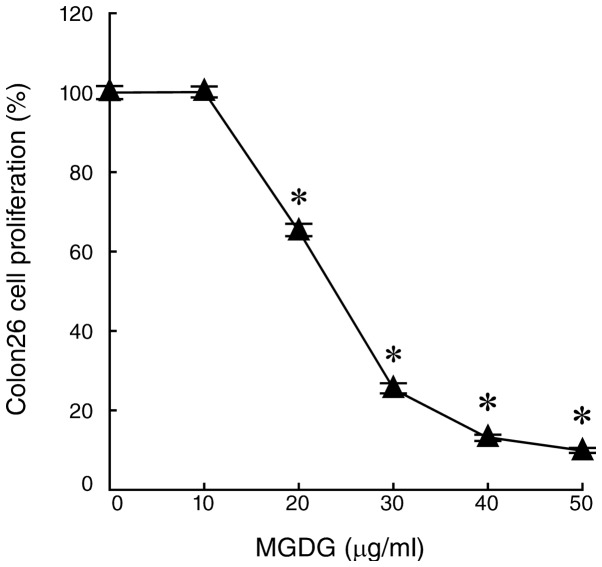
Dose-response curve of purified MGDG from spinach on the cell growth inhibition of the cultured mouse colon cancer cell line, Colon26. Colon26 cells were cultured for 24 h in media containing the indicated concentrations of MGDG. Cell proliferation was determined using the MTT assay (19). All values are expressed as the mean ± SE of five independent experiments. MGDG, monogalactosyl diacylglycerol; MTT, 3-(4,5-dimethylthiazol-2-yl)-2,5-diphenyl-2H-tetrazolium bromide.

First, we investigated the effects of purified MGDG from spinach (Fig. 1) on the cell growth suppression of a mouse colon cancer cell line, Colon26, *in vitro*. These cultured cells were tested using an MTT assay following incubation with 0, 10, 20, 30, 40 or 50 *μ*g/ml MGDG for 24 h. MGDG significantly inhibited the proliferation of the Colon26 cells in a dose-dependent manner and the LD_50_ value was 24 *μ*g/ml (Fig. 2). Previously, we reported that MGDG suppressed the human gastric cancer cell line NUGC-3 to almost the same extent as the Colon26 cell line (7). These results indicate that MGDG is an effective inhibitor of colon cancer cell growth.

### Effect of CD-MGDG complex on in vivo tumor graft growth in mice

**Figure 3:**
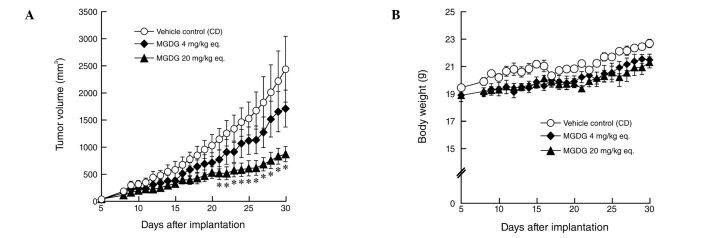
Effect of CD-MGDG complex on tumor growth in mice. (A) Mice with Colon26 solid tumors were orally administered CD (200 mg/kg) as a control group (vehicle control, n=6), CD (40 mg/kg)-MGDG (4 mg/kg) complex (n=5) or CD (200 mg/kg)-MGDG (20 mg/kg) complex (n=6). (B) Body weight curves of mice. All data are expressed as the mean ± SE. ^*^Significantly different from the control, P<0.05. CD, γ-cyclodextrin; MGDG, monogalactosyl diacylglycerol.

The Colon26 mouse colon cells (1×10^6^) were inoculated s.c. into mice. Five days later, the mice received 200 mg/kg CD alone (control), CD (40 mg/kg)-MGDG (4 mg/kg) complex or CD (200 mg/kg)-MGDG (20 mg/kg) complex daily for 26 days. As shown in Fig. 3A, tumors in the control group continued to grow rapidly. By contrast, in the mice treated with purified MGDG from spinach, tumor growth was suppressed in a dose-dependent manner. Treatment of the mice with 4 and 20 mg/kg MGDG eq. inhibited Colon26 tumor growth by 29.8 (P>0.1) and 64.2% (P<0.05), respectively, relative to vehicle-treated controls. In the present study, mice treated with MGDG appeared healthy and showed no marked weight loss compared with vehicle-treated controls (Fig. 3B). Histopathological analysis showed that the lungs, heart, spleen, stomach, liver, pancreas, kidney, intestine and brain of MGDG-treated mice were normal. These findings suggest that MGDG did not have side effects, including mortality or evident toxicity, loss of body weight and/or major organ damage, in mice.

### Effect of CD-MGDG complex on cell proliferation of mouse colon tumor tissue in vivo

To assess the *in vivo* effect of CD-MGDG complex consumption on the proliferation of tumor tissue in mice, the samples were analyzed by H-E staining and PCNA immunostaining. Qualitative histopathologic analysis of H-E-stained sections revealed a substantial decrease in the number of mitotic cells in the tumor tissue of the MGDG-treated mice compared with that of the vehicle-treated controls (Table 1; P<0.05 for 20 mg/kg MGDG eq.). Qualitative histopathological analysis of PCNA-stained sections revealed a decrease in the number of PCNA-positive cells in the tumor tissue of the MGDG-treated mice compared with that of the vehicle-treated controls (Table I; P<0.05 for each dosage). Compared with tumors from mice receiving vehicle alone, the number of mitotic cells and percentage of PCNA-positive cells were reduced by 38.7 and 22.9%, respectively, in the tumors from mice that received 20 mg/kg MGDG eq. These results suggest that orally administered MGDG prevented the mitotic cell proliferation of Colon26 solid tumors in mice in a dose-dependent manner.

### Immunofluorescence staining for CD31 in colon tumor tissue from mice

**Figure 4:**
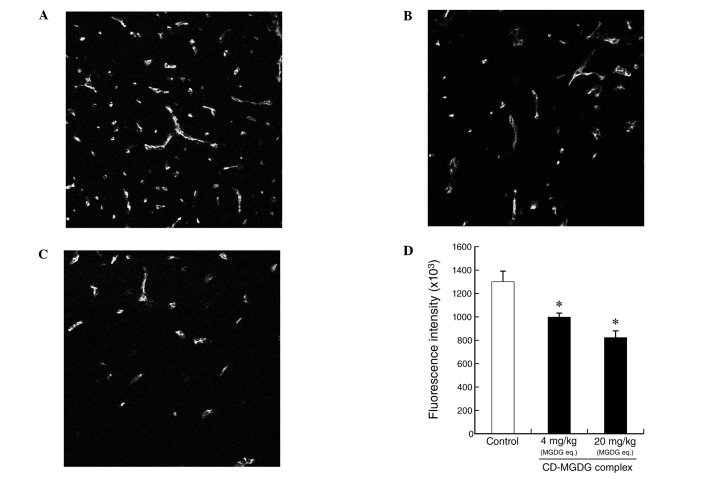
Immunofluorescence staining for CD31 in Colon26 solid tumor tissue. Groups of mice were treated with (A) CD (200 mg/kg) as a control group (vehicle control, n=6), (B) CD (40 mg/kg)-MGDG (4 mg/kg) complex (n=6) or (C) CD (200 mg/kg)-MGDG (20 mg/kg) complex (n=5). (D) Mean fluorescence intensity values of CD31. ^*^Significantly different from the control, P<0.05. CD, γ-cyclodextrin; MGDG, monogalactosyl diacylglycerol.

To evaluate the anti-angiogenesis effect *in vivo*, MGDG was evaluated for dose-dependent modulation of microvessels by immunostaining for CD31. As shown in Fig. 4A–C, a dose-dependent reduction in the staining intensity of the CD31-positive endothelial cells by MGDG was observed at doses of 4 and 20 mg/kg/day in Colon26 solid tumor tissue, using a microscope equipped for immunofluorescence analysis. Mice treated with 4 mg/kg and 20 mg/kg MGDG eq. had fluorescence intensities of 999±35×10^3^ (P<0.05) and 824±58×10^3^ P<0.05) and inhibited CD31 expression by 23.2 and 36.7%, respectively, compared with the vehicle control (1301±91×10^3^; Fig. 4D). The typical averages indicate that MGDG prevented not only CD31-positive endothelial cell expression intensity but also microvessel formation (Fig. 4A–C).

### Effect of CD-MGDG complex on apoptosis induction of colon tumor tissue in mice

The histopathologist randomly observed tumor tissue sections with H-E staining. The number of apoptotic cells tended to increase as the dose of MGDG increased (data not shown). We used a TUNEL technique to detect apoptotic cells in Colon26 tumor tissue sections. The number of TUNEL-positive apoptotic cells was increased in the tumors from the mice treated with MGDG in a dose-dependent manner compared with that in tumors from mice treated with the vehicle (Table II). These data indicate that MGDG administration effectively induces apoptosis in tumor cells *in vivo*, as has been demonstrated *in vitro* in a previous study (7).

## Discussion

In the current study, we evaluated the efficacy of oral CD-MGDG complex administration for the treatment of implanted solid tumors in mice. CD is able to render fat-soluble materials water-soluble, therefore, the CD-MGDG complex may be useful as an anticancer functional food and/or drug. CD itself is digested in the body and is a safe agent (20). In the *in vitro* cultured cancer cell growth assay, MGDG alone (without CD) significantly suppressed the proliferation of colon cancer cells (Fig. 2). The vehicle control (CD alone) had no effective antitumor activity (Fig. 3A), therefore, purified MGDG from spinach may be effective. These results show that MGDG inhibits tumor growth in a dose-dependent manner and immunohistochemical analysis suggests that the antitumor efficacy of MGDG may be associated with anti-angiogenetic, anti-proliferative and apoptotic effects in tumor tissue without adverse health effects.

MGDG is a non-nutrient compound contained in vegetables, grains and fruits. MGDG content differs among plants (12) and is ingested daily in food. The chemical structure of MGDG comprises two acyloxy groups (R_1_ and R_2_ in Fig. 1) derived from fatty acid molecules. In the present study, we used spinach MGDG which is rich in n-3 α-linolenic acid (26.3% of the total fatty acids in spinach MGDG) (7). MGDG from wheat flour includes non-n-3 fatty acids, such as linoleic acid, which is an n-6 fatty acid, and saturated fatty acids (21). However, the fatty acid composition influences the antitumor effect (22). Therefore, these findings suggest that researchers should observe and note the lipid contents and fatty acid composition in MGDG studies.

As few studies have investigated MGDG, it is unknown whether MGDG is absorbed. Previously, we reported that mammalian lipase is able to hydrolyze some MGDG into monogalactosyl monoacylglycerol (MGMG) *in vitro*(15). A study of orally administered MGDG *in vivo* revealed that it is digested to MGMG and monogalactosyl glycerol (MGG) by a digestive enzyme and enterobacteria, respectively, and MGG is not absorbed (21). However, the oral administration of MGDG demonstrated strong and dose-dependent inhibition of tumor growth in a mouse model in the current study. These results suggest that part of the MGDG compound, such as MGMG and/or MGG, was absorbed and re-synthesized or that undifferentiated MGDG entered the blood stream. In addition, these results suggest that MGDG is not completely degraded and possesses antitumor activity, since components of MGDG, galactose and glycerol, do not have an antitumor effect, and fatty acids have a weak antitumor effect (23,24).

As shown in Tables I and II and Fig. 4, we analyzed tumor tissue following the administration of MGDG to mice. These results show that MGDG prevented tumor growth by inhibiting angiogenesis and and reduced the count of cells that stained positive for PCNA, which is a proliferation marker (25,26), and was accompanied by an increase of apoptosis. Certain studies have suggested that replicative DNA polymerases (α, δ and ε) and tumor angiogenesis have potential as cancer therapeutic targets (27–31). In particular, replicative DNA polymerases are essential for cancer cell proliferation. In addition, tumor growth depends on angiogenesis, since tumors have to be located within 200 *μ*m of blood vessels to obtain nutrients and oxygen (32). We previously observed that MGDG from spinach inhibited the activities of mammalian replicative DNA polymerases, but had no inhibitory effect on other mammalian DNA polymerases, including repair-related β, η, ι, κ, λ and *μ*, and MGDG suppressed human umbilical vein endothelial cells (HUVEC) tube formation, HUVEC proliferation and tumor angiogenesis *in vitro* and *ex vivo*(16,17). However, the data in Fig. 2 and Table I suggest that the antitumor effects of MGDG are not entirely dependent on angiogenesis. The present *in vitro* study did not include endothelial cells and it appears that MGDG has a direct effect on tumor cells.

In conclusion, the present study showed that the natural product MGDG obtained from vegetables, fruits and grains is safe with a potent oral antitumor effect, including anti-proliferative, anti-angiogenesis and apoptosis-inducing activity. Our results suggest that MGDG from food has cancer-preventive and health-promotion effects.

## Figures and Tables

**Table I:** Counts of mitotic and PCNA-positive cells in Colon26 tumor sections.

Variable	Mitosis (/HPF)	PCNA (%)
Vehicle control (CD)	19.1±1.6	77.0±1.4
CD-MGDG complex (MGDG 4 mg/kg eq.)	14.7±1.5	67.3±2.2^a^
CD-MGDG complex (MGDG 20 mg/kg eq.)	11.7±1.3^a^	59.4±2.5^a^

Mice were orally treated with CD (200 mg/kg), CD (40 mg/kg)-MGDG (4 mg/kg) complex or CD (200 mg/kg)-MGDG (20 mg/kg) complex. Histopathological analysis was performed at a HPF (×400). Data are expressed as the mean ± SE.

a Significantly different from the control (P<0.05). CD, γ-cyclodextrin; MGDG, monogalactosyl diacylglycerol; HPF, high power field; PCNA, proliferating cell nuclear antigen.

**Table II:** TUNEL analysis of Colon26 tumor sections.

Variable	TUNEL-positive cells (/HPF)
Vehicle control (CD)	5.2±0.9
CD-MGDG complex (MGDG 4 mg/kg eq.)	9.0±0.8
CD-MGDG complex (MGDG 20 mg/kg eq.)	13.7±1.8^a^

Mice were orally treated with CD (200 mg/kg), CD (40 mg/kg)-MGDG (4 mg/kg) complex or CD (200 mg/kg)-MGDG (20 mg/kg) complex. TUNEL analysis was performed at a HPF (400×). Data are expressed as the mean ± SE.

a Significantly different from the control (P<0.05). CD, γ-cyclodextrin; MGDG, monogalactosyl diacylglycerol; HPF, high power field; TUNEL, terminal deoxynucleotidyl transferase dUTP nick-end labeling.

## Abbreviations:

MGDG: monogalactosyl diacylglycerol
CD: cyclodextrin
PCNA: proliferating cell nuclear antigen
TUNEL: terminal deoxynucleotidyl transferase dUTP nick-end labeling
SQDG: sulfoquinovosyl diacylglycerol
DGDG: digalactosyl diacylglycerol
MTT: 3-(4,5-dimethylthiazol-2-yl)-2,5-diphenyl-2H-tetrazolium bromide
H-E: hematoxylin and eosin
HUVEC: human umbilical vein endothelial cell
